# Thoracic aortic pseudoaneurysm with esophageal compression due to brucellosis: a rare case report

**DOI:** 10.1186/s12879-026-13955-7

**Published:** 2026-07-07

**Authors:** Pei Gu, Meina Lang, Rongxia Sang, Shumei Zhang, Run Liu, Nan Liu, Xin Zhao, Zhengrong Guo, Yulan Liu

**Affiliations:** Department of Gastroenterology, Shijiazhuang People’s Hospital, No. 365, Jianhua South Street, Yuhua District, Shijiazhuang City, Hebei Province 050000 China

**Keywords:** Brucella, Pseudoaneurysm, Dysphagia, Case report

## Abstract

**Background:**

Brucellosis can affect multiple systems throughout the body, with cardiovascular involvement being extremely rare. This patient initially presented with dysphagia, lacking typical symptoms such as “undulant fever”, lymphadenopathy, or joint and muscle pain. The final diagnosis was a Brucella-infected pseudoaneurysm of the thoracic aorta, which, to our knowledge, is the first reported case of its kind.

**Case presentation:**

A 66-year-old Chinese male patient with no history of thoracic aortic surgery and no history of blunt chest trauma, was admitted to the hospital because of dysphagia more than 10 days. Gastroscopy revealed a raised lesion measuring approximately 2 × 2 cm in size 32–34 cm from the incisors, with a normal mucosal surface. The patient had elevated white blood cell count and CRP levels. Endoscopic ultrasound revealed a heterogeneous echoic mass, and pathological examination of the puncture sample revealed abundant neutrophils. Contrast-enhanced chest CT indicated a pseudoaneurysm of the thoracic aorta. The results of both the Rose Bengal plate test and the tube agglutination test were both positive, confirming that the thoracic aortic pseudoaneurysm was caused by Brucella infection, which compressed the esophagus. The patient was treated with antibiotics, and thoracic aortic endovascular stent graft placem ent was planned after infection control. However, one month later, the patient developed hematemesis, and surgical intervention was urgently recommended. The family refused further treatment, and the patient was discharged. Approximately two weeks after discharge, the patient passed away at home.

**Conclusion:**

Brucella infection involving the cardiovascular system can lead to the extremely rare complication of a thoracic aortic pseudoaneurysm. This condition is associated with a high risk of rupture and life-threatening hemorrhage, resulting in an extremely poor prognosis. In patients who present with an infectious pseudoaneurysm, Brucella infection should be considered in the differential diagnosis to reduce the risks of misdiagnosis and missed diagnosis.

## Background

Brucellosis is a zoonotic disease caused by Brucella infection.Brucella are gram-negative, short coccobacilli, obligate aerobic bacteria that commonly parasitize livestock such as sheep and cattle. In China, the primary transmission route is through sheep. Recent studies indicate that the global incidence of brucellosis is higher than previously estimated, with 1.6–2.1 million human cases annually.Given the factors of missed diagnoses and misdiagnoses, the actual disease burden may be higher than is currently estimated, making it a significant threat to global public health security [1].

Brucellosis can affect multiple organs and systems, such as the liver, spleen, lymph nodes, osteoarticular system, genitourinary system, central nervous system, and cardiovascular system. Cardiovascular involvement, although rare, primarily manifests as arterial and venous thrombosis, vasculitis, and endarteritis, potentially leading to peripheral or cerebrovascular aneurysms—a major cause of mortality in patients with brucellosis [2]. Brucella-infected pseudoaneurysms may occur in any aortic segment (ascending, arch, descending, or abdominal aorta) with progress rapidly, and are at high risk of rupture [3].

Given the scarcity of reported cases, such pseudoaneurysms are frequently missed. This article presents a rare case of a thoracic aortic pseudoaneurysm with esophageal compression caused by brucellosis, with the aim of increasing clinical awareness, guiding diagnosis and treatment, and preventing diagnostic errors.

## Case presentation

**Fig. 1:**
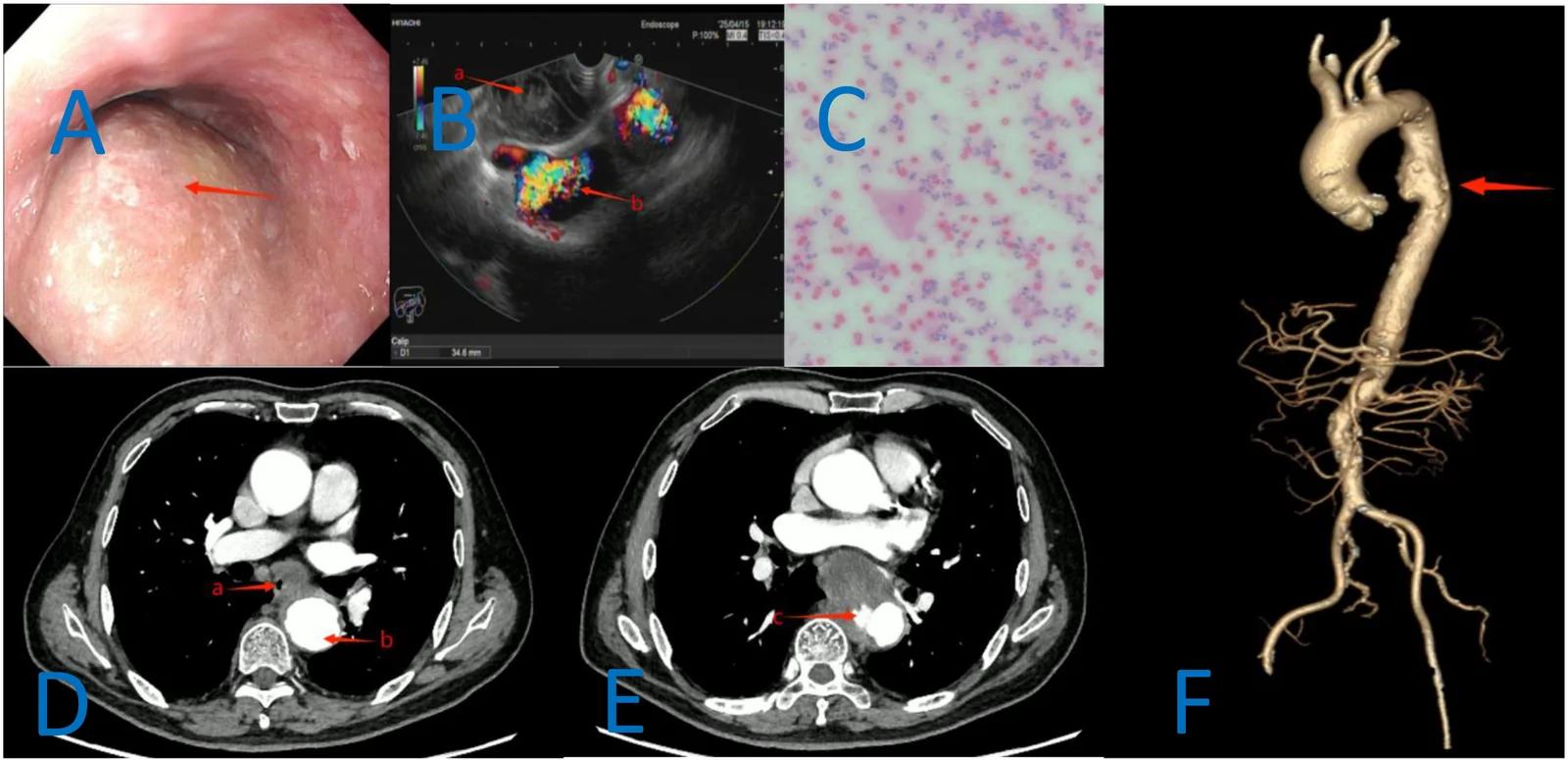
(**A**) Endoscopic image of the esophagus. The arrow indicates the mass protruding into the esophageal lumen. (**B) **Endoscopic ultrasonography(EUS) scan of the esophagus.The arrow labeled “a” points to the area of heterogeneous echogenicity.The arrow labeled “b” indicates the thoracic aorta. (**C) **Pathological examination of the ultrasound endoscopy-guided fine-needle aspiration (EUS-FNA) specimen reveals a hemorrhagic background containing numerous neutrophils and scant squamous epithelial cells. (**D**, **E) **Two chest CT scan images. The arrow labeled “a” indicates the compressed esophagus. The arrow labeled “b” indicates marks the thoracic aorta. The arrow labeled “c” indicates the pseudoaneurysm. (**F)**Computed tomography angiogram(CTA) of the patient’s thoracoabdominal aorta. The arrow indicates the thoracic aortic pseudoaneurysm (note the saccular outpouching with irregular wall morphology)

A 66-year-old male patient from Shijiazhuang, with a history of type 2 diabetes mellitus, coronary heart disease (CHD), and hypertension, as well as a history of smoking and alcohol consumption, with no history of thoracic aortic surgery and no history of blunt chest trauma, was admitted to the hospital because of dysphagia more than 10 days. was admitted to the Department of Gastroenterology at Shijiazhuang People’s Hospital on April 11, 2025, due to dysphagia persisting for more than 10 days. Physical examination revealed no significant abnormalities.Outpatient gastroscopy revealed a 2 × 2 cm submucosal bulge at 32–34 cm from the incisors, with intact overlying mucosa (Fig. 1 A).

Laboratory tests at admission revealed elevated inflammatory markers: the C-reactive protein (CRP) concentration was 114.23 mg/L, the white blood cell (WBC)count was 11.98 × 10⁹/L and the neutrophil count was 6.91 × 10⁹/L. The transthoracic echocardiography (TTE) findings were normal.Endoscopic ultrasonography (EUS) revealed a lesion that exhibited heterogeneous echogenicity, contained an anechoic structure within it, and was surrounded by a capsule. The lesion was in close proximity to the esophageal muscularis propria, measured approximately 5 × 4 cm in size, and demonstrated minimal blood flow on Doppler imaging (Fig. 1B). Pathological examination revealed scattered neutrophils and inflammatory cells without malignant cells (Fig. 1 C). Contrast-enhanced chest CT showed an irregular thoracic aortic intima and extravasation of high-density contrast medium, both of which are consistent with a thoracic aortic pseudoaneurysm (Fig. 1D, E).

The patient reported no history of recurrent oral or genital ulcers. The pathergy test (skin needle test) was negative, and autoimmune antibody testing was negative. Therefore, autoimmune diseases were considered unlikely. The patient had no history of tuberculosis (TB) or known TB exposure. The purified protein derivative (PPD) skin test was negative, and pathological examination of the aspirated material was negative for acid-fast bacilli on staining. These findings do not support the presence of Mycobacterium tuberculosis infection. Further history-taking revealed that although the patient was a farmer residing in a nonendemic area, he had raised sheep for more than a decade, frequently assisting in lamb deliveries without protective measures. On the basis of this occupational exposure, additional testing revealed a positive Rose Bengal plate agglutination test and a diagnostic serum agglutination test titer of 1:800 (diagnostic cutoff ≥ 1:50). The patient’s blood cultures were negative for bacterial growth, potentially because of a low bacterial load. Further investigation with ultrasonography of superficial lymph nodes revealed no enlarged lymph nodes. Computed tomography angiography (CTA) of the entire aorta revealed that the involvement was isolated to the thoracic aorta (Fig. 1 F).

The patient was treated with doxycycline 0.1 g bid, rifampin 750 mg qd, and levofloxacin 0.5 g qd for infection control and underwent aggressive blood pressure management. Following treatment, the patient’s inflammatory markers decreased, and dysphagia and chest pain significantly improved. The patient was discharged in a clinically stable condition. Thoracic aortic endovascular stent graft placement was planned after confirmation of infection control.

**Fig. 2:**
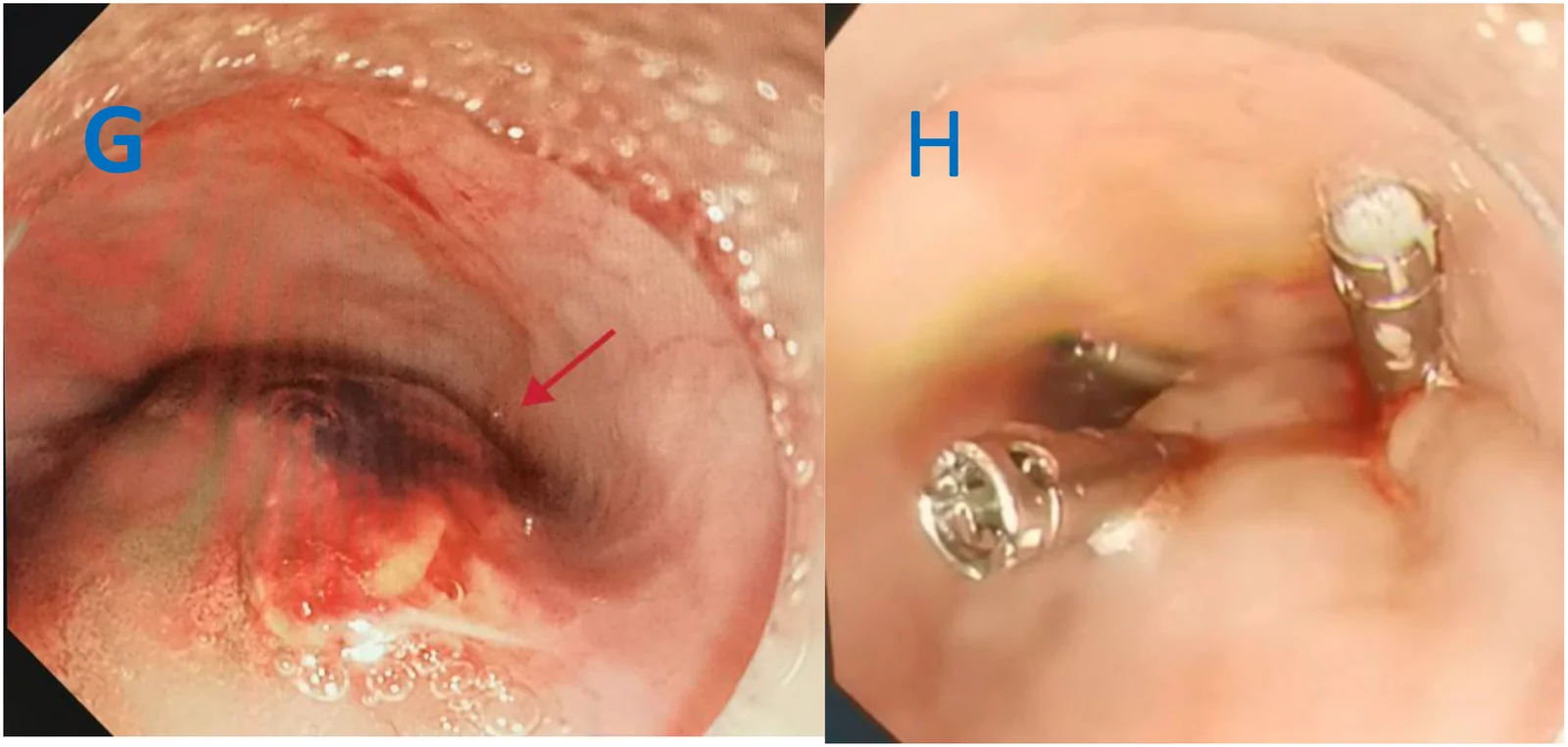
This is an endoscopic image from the patient’s emergency procedure. (**G**) The arrow indicates erosive and bleeding mucosa at the site of esophageal compression. (**H**) the patient is shown undergoing hemostatic treatment with titanium clip closure

Following discharge, the patient remained clinically stable. The Brucella agglutination test was repeated on May 20, 2025 and revealed a decreased titer of 1:400. However, on May 21, 2025, the patient was readmitted because of “hematemesis for 3 hours”. With massive hemorrhage and unstable vital signs, emergency gastroscopy revealed mucosal erosion and active bleeding at the site of the original esophageal protrusion (Fig. 2G). Bleeding was controlled with titanium clip placement, after which no further hematemesis occurred (Fig. 2 H). Urgent surgical intervention was strongly recommended, but the family declined and the patient was discharged. During follow-up, the family reported that the patient passed away on June 13, 2025; further details are unknown.

## Discussion

Brucellosis is a zoonotic infectious disease. The general population is universally susceptible. However, compared with the general population, groups such as farmers, herdsmen, veterinarians, tannery workers, and slaughterhouse employees have higher infection rates.This elevated risk is likely due to high-risk behaviors, such as unprotected contact with livestock birth secretions and contaminated materials during work, which increase the likelihood of Brucella infection [4].

Considering that Brucellosis can affect multiple organ systems and its clinical presentation and severity can vary, its diagnosis on the basis of any single symptom is challenging. Bacterial invasion and endotoxins play a dominant role in the acute phase, whereas delayed hypersensitivity reactions and granuloma formation characterize the chronic phase [5].The diagnosis of this case was challenging because the patient lacked typical symptoms such as fever, chills, myalgia, or lymphadenopathy, but instead presented with dysphagia as the initial symptom.

Cardiovascular involvement in brucellosis is rare, with only a few reported cases in the past decade [6–10], occurring in only 3% of cases [9, 11]. A meta-analysis was done in Turkey, evaluating 4204 brucellosis patients reported IE in 1.2% of cases [7]. According to the review by Willems SA et al. [11], over the past seven decades, there were 71 patients who developed cardiovascular complications, with an overall death rate of 22%. Most patients were male (86%), and 66% had a known history of exposure to Brucella. Around one quarter (23%) acquired the infection while traveling in a region where the disease is highly or moderately endemic. Nearly half of the infections involved the abdominal aorta (49%), followed by the ascending (37%) and descending (13%) parts of the thoracic aorta. The most common findings were infected aneurysms (61%) and ulcerative lesions (16%). Aortic rupture was documented in 31% of cases, occurring mainly in the abdominal (49%) and descending thoracic aorta (44%). The majority of patients (59%) were treated with open surgery combined with a prolonged course of antibiotics. Over the last 15 years, there has been a noticeable shift toward endovascular approaches.

However, it has exceptionally high mortality [11–12].Aortic manifestations primarily include pseudoaneurysm formation. Following infection of compromised vasculature by Brucella organisms, the bacteria upregulate endothelial adhesion molecules and secrete proinflammatory chemokines, thereby triggering a severe localized inflammatory cascade. This leads to endothelial injury with exposure of the tunica media, structural damage to the aortic wall, and transmural hemorrhage. The extravasated blood becomes encapsulated by the surrounding soft tissue, resulting in the formation of a hematoma with persistent communication between the hematoma cavity and aortic lumen, resulting in a pseudoaneurysm [12–13]. Pseudoaneurysms are nonself-limiting lesions with significant risks of rupture and life-threatening hemorrhage. A contained rupture may temporarily occur, but once complete rupture occurs, mortality approaches 100% because of catastrophic exsanguination. According to prior studies, male sex, advanced age, smoking status, white ethnicity, a family history of abdominal aortic aneurysm and atherosclerotic disease constitute the primary positive correlates for pseudoaneurysm development. According to the literature, additional documented risk factors include immunocompromised states (e.g., diabetes mellitus, corticosteroid therapy, and chemotherapy), preexisting vascular injury, and chronic alcohol abuse [14–15]. This patient’s demographic and clinical profile revealed advanced age, male sex, zoonotic exposure, and cumulative cardiovascular risk factors (tobacco use, hypertension, diabetes), placing him in the highest risk stratum for severe brucellosis manifestations.

The diagnostic workup for suspected brucellosis must include serology, blood cultures and epidemiological risk assessment (occupational/livestock exposure). Imaging studies are particularly critical for the diagnosis of this condition. In patients with infectious thoracic aortic pseudoaneurysms, arterial CTA typically reveals a hypodense soft tissue mass surrounding the artery that exhibits patchy or annular enhancement. Adjacent structures such as the kidneys or vertebral bodies may undergo destructive changes. The pseudoaneurysm often presents as an eccentric, sac-like outpouching with a lobulated internal architecture, containing irregular filling defects and gas bubbles within the lumen, while wall calcification is rarely observed. Notably, 47–73% of infectious abdominal aortic pseudoaneurysms also exhibit signs of rupture [16]. The presence of gas within the pseudoaneurysm or surrounding tissues strongly corroborates the diagnosis of an infected pseudoaneurysm [11].In summary, aortic CTA provides a comprehensive and precise evaluation of lesion location and severity, serving as a critical reference for therapeutic decision-making.

Upon confirmation of Brucella infection, prompt initiation of combination antibiotic therapy is imperative. The treatment principles include early intervention, combination drug therapy, adequate dosing, and a complete course that can be extended when clinically indicated. Monotherapy is insufficient to control Brucella infection and is associated with significantly increased recurrence rates [17].Tetracyclines and rifamycins are commonly employed, while fluoroquinolones, sulfonamides, aminoglycosides, and third-generation cephalosporins may also be considered [18].

Evidence confirms that the levofloxacin-rifampin-doxycycline regimen demonstrates definitive efficacy in brucellosis management, offering the dual advantages of accelerated symptom resolution and favorable safety profiles, warranting broader clinical adoption.

Studies indicate that the rupture rate following conservative management alone is as high as 53% [19]. For infectious aortic pseudoaneurysms, endovascular stent graft placement yields favorable prognostic outcomes [9, 15, 20]. In this patient, thoracic aortic endovascular stent graft implantation was planned following aggressive antimicrobial therapy. Unfortunately, the lesion eroded into the esophagus, resulting in massive gastrointestinal hemorrhage. Although endoscopic hemostasis was achieved with titanium clip placement, this intervention could not alter the ultimately dismal prognosis.

The databases used in this review were PubMed and CNKI, covering the period from 2007 to 2025, with the search keywords “Brucella” and “pseudoaneurysm”.

## Conclusion

This case presented significant diagnostic challenges. Given that dysphagia was the initial manifestation in the absence of classic Brucella infection symptoms, it was prone to misdiagnosis as a mediastinal tumor or esophageal carcinoma. Serological, microbiological, and imaging studies are necessary for definitive differentiation.Furthermore, pseudoaneurysms are at high risk of rupture and fatal hemorrhage, portending a dismal prognosis. In patients with infectious pseudoaneurysms, the active exclusion of Brucella infection is imperative to reduce the risk of misdiagnosis.

## Data Availability

All data and materials generated or analysed during this study are included in this published article.

## Abbreviations

CRP: The C-reactive protein
CHD: Coronary heart disease
WBC: The white blood cell
TTE: The transthoracic echocardiography
EUS: Endoscopic ultrasonography
FNA: fine-needle aspiration
TB: Tuberculosis
PPD: The purified protein derivative
CTA: Computed tomography angiography
